# The Role of PD-L1 in Treatment Decision-Making for Perioperative *EGFR*-Mutated Lung Cancer and its Genomic Analysis

**DOI:** 10.1245/s10434-026-19781-0

**Published:** 2026-05-27

**Authors:** Yi-Fan Qi, Zhen-Bin Qiu, Wei-Ye Huang, Chao Zhang, Rui Fu, Guang-Ling Jie, Xiong-Wen Yang, Yi-Duo Lin, Hong-Ji Li, Qian Cui, Jian Su, Zhi Xie, Ben-Yuan Jiang, Xu-Chao Zhang, Xue-Ning Yang, Yi-Long Wu, Wen-Zhao Zhong

**Affiliations:** 1 Guangdong Lung Cancer Institute, Guangdong Provincial People’s Hospital, Guangdong Academy of Medical Sciences, Southern Medical University, Guangzhou, China; 2 School of Medicine, South China University of Technology, Guangzhou, China; 3 Department of Pathology, Guangdong Provincial People’s Hospital, Guangdong Academy of Medical Sciences, Southern Medical University, Guangzhou, China; 4 Shanghai Lung Cancer Center, Shanghai Chest Hospital, Shanghai Jiao Tong University School of Medicine, Shanghai, China; 5 Guangdong Provincial Key Laboratory of Translational Medicine in Lung Cancer, Guangdong Provincial People’s Hospital, Guangdong Academy of Medical Sciences, Southern Medical University, Guangzhou, China

**Keywords:** Programmed death-ligand 1, Epidermal growth factor receptor, Adjuvant therapy, Neoadjuvant therapy, Immunochemotherapy, Non-small cell lung cancer

## Abstract

**Background:**

Epidermal growth factor receptor-mutated (*EGFR*-M+) non-small-cell lung cancer (NSCLC) represents a substantial proportion of lung cancer and remains associated with a considerable risk of postoperative recurrence despite advances in targeted therapies. The role of programmed death-ligand 1 (PD-L1) expression in the perioperative treatment of *EGFR*-M+ NSCLC remains controversial. The gene mutation landscape of PD-L1 in *EGFR*-M+ NSCLC and its relationship with prognosis remain poorly understood.

**Methods:**

We retrospectively included patients with *EGFR*-M+ NSCLC, including 43 patients with stage IB–IV disease who underwent immunotherapy combined with chemotherapy followed by surgery (NeoGroup) and 499 patients with stage IB–III disease undergoing surgery (AdjGroup). Disease-free survival, radiological response, pathological response, clinicopathological factors, and genomic characteristics were analyzed to evaluate their associations with PD-L1 tumor proportion score (TPS).

**Results:**

The NeoGroup had objective response, pathological complete response, and major pathologic response rates of 44.2%, 18.6%, and 41.9%, respectively. A higher frequency of PD-L1 TPS ≥1% at baseline correlated with better radiological responses. Patients who had a major pathologic response also had a greater increase in PD-L1 TPS from baseline after therapy (*P* = 0.0195). In the AdjGroup (N = 499), PD-L1 TPS ≥50% independently predicted shorter disease-free survival. Patients with PD-L1 ≥1% more frequently harbored *TP53* co-mutations and had poorer survival in external cohorts.

**Conclusion:**

PD-L1 plays a dual role in perioperative *EGFR*-M+ NSCLC, indicating higher recurrence risk after surgery while predicting improved response to immunochemotherapy. PD-L1 may serve as a biomarker to guide individualized perioperative treatment strategies in *EGFR*-M+ NSCLC. Prospective validation is still warranted.

**Supplementary Information:**

The online version contains supplementary material available at https://doi.org/10.1245/s10434-026-19781-0.

Adjuvant chemotherapy provides limited benefit in non-small-cell lung cancer (NSCLC), with an approximately 5% improvement in 5-year overall survival (OS).^1^ Epidermal growth factor receptor-tyrosine kinase inhibitors (EGFR-TKIs) have significantly improved the outcomes of patients with *EGFR*-mutated (*EGFR*-M+) NSCLC.^2–6^ However, early recurrence of lung cancer highlights the need to identify factors associated with recurrence and prognosis in patients with *EGFR*-M+ NSCLC.^7,8^

Immune checkpoint inhibitors (ICIs) have revolutionized the treatment paradigm, especially for *EGFR*-wildtype NSCLC.^9–12^ Programmed death-ligand 1 (PD-L1) is a predictive biomarker of ICI efficacy.^13^ However, its role in *EGFR*-M+ NSCLC remains controversial.^9,14^ In patients with advanced *EGFR*-M+ NSCLC, PD-L1 expression is associated with a poor prognosis or resistance to TKIs.^15–17^ However, the FLAURA study found that PD-L1 status did not affect osimertinib efficacy in advanced *EGFR*-M+ NSCLC.^18^ In perioperative *EGFR*-M+ NSCLC, prospective data on the impact of PD-L1 on TKI efficacy and long-term patient outcomes are limited. Small retrospective *EGFR* mutant cohorts link PD-L1 ≥1% to shorter disease-free survival (DFS) and OS, but their prognostic value in stage IB–III *EGFR*-M+ NSCLC remains unclear.^19^

*EGFR*-M+ NSCLC often exhibits a “cold” immune microenvironment with limited benefits from ICIs,^20–22^ possibly due to lower PD-L1 levels or low tumor mutational burden (TMB).^20,23^ Immunotherapy can be effective for patients with NSCLC with PD-L1 expression or other favorable immune features. Neoadjuvant immunotherapy with chemotherapy may induce a favorable tumor response, even in oncogene-driven NSCLC.^24–26^ The PEARLS/KEYNOTE-091 trial reported an improved DFS with adjuvant pembrolizumab in stage IB–IIIA *EGFR*-M+ NSCLC after chemotherapy, highlighting the possible benefit of ICIs in the *EGFR*-M+ subgroup.

PD-L1 is heterogeneously associated with survival outcomes in patients with *EGFR*-M+ NSCLC treated with EGFR-TKIs or other therapies, and its prognostic value in early-stage, resectable *EGFR*-M+ NSCLC remains unclear.^15–19^ PD-L1 assessment is affected by assay-related variability and intratumoral heterogeneity, which may contribute to discrepant results across studies and cut-offs.^27–29^

Clinicogenomic analyses have suggested that oncogene-driven NSCLC exhibits distinct immune-related genomic features, and oncogene-specific differences in PD-L1 expression, TMB, and immunotherapy outcomes.^21,22^ The genomic landscape associated with PD-L1 expression in perioperative *EGFR*-M+ NSCLC, and its relationship with clinical outcomes, remains poorly understood. Additionally, dynamic changes in PD-L1 during perioperative treatment and their association with radiological and pathological responses have not been systematically explored in such cases.

This study aimed to comprehensively evaluate the clinical and genomic significance of PD-L1 in perioperative *EGFR*-M+ NSCLC. We assessed (i) the prognostic value of PD-L1 tumor proportion score (TPS) in resected *EGFR*-M+ NSCLC managed without immunotherapy, (ii) the association of baseline and dynamic PD-L1 changes with radiological and pathological responses in patients receiving induction immunochemotherapy, and (iii) the genomic characteristics associated with PD-L1 expression, including co-mutation patterns and their prognostic relevance in external cohorts.

## Patients and Methods

### Study Design and Patients

We screened patients with NSCLC at Guangdong Provincial People's Hospital (GDPH) between 2008 and 2024 and confirmed *EGFR* mutations using next-generation sequencing or an amplification-refractory mutation system. Patients harboring common *EGFR* mutations (exon 19 deletion [19Del] or L858R) or other rare *EGFR* mutations were included. Those with exon 19Del or L858R mutations accompanied by other *EGFR* mutations were classified as exon 19Del or L858R, respectively.

Participants were divided into two cohorts. The AdjGroup consisted of patients with pathological stage IB–IIIB *EGFR*-M+ NSCLC who underwent R0 resection, with or without adjuvant therapies. Patients with a history of other malignancies or missing *EGFR* mutation data were excluded. The NeoGroup included patients with stage IB–IIIA *EGFR*-M+ NSCLC who received neoadjuvant therapy and patients with stage IIIB–IVB *EGFR*-M+ NSCLC who received conversion therapy to enable surgery. All patients in the NeoGroup received immunotherapy and chemotherapy before surgery.^30^

### External Cohorts

External cohort 1 involved patients with *EGFR*-M+ lung adenocarcinoma from The Cancer Genome Atlas (TCGA-LUAD, *N* = 73) and was used to validate the impact of genomic features on survival. External cohort 2 was an independent cohort of patients with stage I–III *EGFR*-M+ lung adenocarcinoma (CELL-LUAD, *N* = 43).^31^ These external cohorts provided additional evidence on the prognostic significance of the molecular features discovered in our cohorts.

### Immunohistochemistry for PD-L1

PD-L1 expression in tumors was evaluated by immunohistochemistry. Formalin-fixed, paraffin-embedded (FFPE) tumor specimens were stained predominantly with monoclonal anti-PD-L1 antibody (clone 22C3, Dako, Agilent Technologies, Santa Clara, CA, USA). TPS was defined as the percentage of viable tumor cells showing membranous PD-L1 staining and was categorized as follows: TPS ≥50%, high expression; TPS 1–49%, intermediate expression; TPS ≥1%, positive expression; and TPS <1%, negative.^32,33^ A minority of tumor specimens were stained with SP142/ZR3 (Table 1).

**Table 1:** Characteristics of patients with *EGFR*-mutated non-small-cell lung cancer (NSCLC) undergoing direct surgery (AdjGroup)

Category	Details	Overall	PD-L1 <1%	1% ≤ PD-L1 <50%	PD-L1 ≥50%	*P*-value
N		499	219	194	59	
Sex	Female	281 (56.31)	127 (57.99)	111 (57.22)	27 (45.76)	0.2258
	Male	218 (43.69)	92 (42.01)	83 (42.78)	32 (54.24)	
Age^a^	Old	282 (56.51)	126 (57.53)	105 (54.12)	36 (61.02)	0.5974
	Young	217 (43.49)	93 (42.47)	89 (45.88)	23 (38.98)	
Smoker	No	425 (85.17)	187 (85.39)	163 (84.02)	49 (83.05)	0.8778
	Yes	74 (14.83)	32 (14.61)	31 (15.98)	10 (16.95)	
Pathological stage	IB	97 (19.44)	60 (27.40)	27 (13.92)	7 (11.86)	**0.0162**
	IIA	11 (2.20)	6 (2.74)	2 (1.03)	1 (1.69)	
	IIB	131 (26.25)	50 (22.83)	60 (30.93)	17 (28.81)	
	IIIA	246 (49.30)	99 (45.21)	100 (51.55)	31 (52.54)	
	IIIB	14 (2.81)	4 (1.83)	5 (2.58)	3 (5.08)	
Adjuvant type	No	113 (22.65)	60 (27.40)	36 (18.56)	13 (22.03)	0.1598
	Chemotherapy	154 (30.86)	68 (31.05)	48 (24.74)	20 (33.90)	
	Targeted therapy	139 (27.86)	56 (25.57)	65 (33.51)	15 (25.42)	
	Other	29 (5.81)	11 (5.02)	15 (7.73)	2 (3.39)	
	Unknown	64 (12.83)	24 (10.96)	30 (15.46)	9 (15.25)	
*EGFR* mutation	19Del	221 (44.29)	90 (41.10)	87 (44.85)	27 (45.76)	0.7432
	L858R	234 (46.89)	111 (50.68)	90 (46.39)	25 (42.37)	
	Other	44 (8.82)	18 (8.22)	17 (8.76)	7 (11.86)	
*EGFR* method	ARMS	332 (69.02)	140 (66.04)	131 (69.68)	42 (72.41)	0.5711
	NGS	149 (30.98)	72 (33.96)	57 (30.32)	16 (27.59)	
Histology	Adenocarcinoma	473 (94.79)	214 (97.72)	186 (95.88)	50 (84.75)	**0.0022**
	Adenosquamous carcinoma	22 (4.41)	5 (2.28)	6 (3.09)	7 (11.86)	
	SARC	1 (0.20)	0 (0.00)	0 (0.00)	1 (1.69)	
	LCNEC	1 (0.20)	0 (0.00)	1 (0.52)	0 (0.00)	
	Squamous	2 (0.40)	0 (0.00)	1 (0.52)	1 (1.69)	
Pathological grade	I	9 (2.22)	6 (3.21)	2 (1.21)	1 (2.63)	**<0.0001**
	II	246 (60.74)	126 (67.38)	101 (61.21)	7 (18.42)	
	III	150 (37.04)	55 (29.41)	62 (37.58)	30 (78.95)	
Pathological subtype (ade=473)^2^	ACN	353 (78.97)	175 (84.54)	142 (78.89)	27 (57.45)	**<0.0001**
	LEP	13 (2.91)	9 (4.35)	3 (1.67)	1 (2.13)	
	MIP	10 (2.24)	3 (1.45)	7 (3.89)	0 (0.00)	
	PAP	20 (4.47)	5 (2.42)	11 (6.11)	1 (2.13)	
	SOL	47 (10.51)	13 (6.28)	16 (8.89)	18 (38.30)	
	SARC	1 (0.22)	0 (0.00)	1 (0.56)	0 (0.00)	
	Mucinous/enteric/colloid	3 (0.67)	2 (0.97)	0 (0.00)	0 (0.00)	
Pleural invasion	No	235 (48.45)	107 (49.77)	80 (42.33)	35 (60.34)	**0.044**
	Yes	250 (51.55)	108 (50.23)	109 (57.67)	23 (39.66)	
Vascular invasion	No	392 (80.82)	183 (84.33)	144 (77.42)	47 (81.03)	0.2095
	Yes	93 (19.18)	34 (15.67)	42 (22.58)	11 (18.97)	
PD-L1^c^		1 (0–10)	0 (0–0)	5 (2–10)	70 (55–90)	**<0.0001**
PD-L1 methods	22C3	442 (94.24)	199 (91.28)	185 (96.35)	58 (98.31)	0.0519
	SP142	26 (5.54)	19 (8.72)	6 (3.12)	1 (1.69)	
	ZR3	1 (0.21)	0 (0.00)	1 (0.52)	0 (0.00)	
TMB^c^		3.995 (2.212–5.675)	3.000 (2.000–5.000)	4.000 (2.990–5.600)	5.865 (4.697–7.075)	**0.0067**

Data are presented as n (%) or median (interquartile range) unless otherwise indicated. Bold values indicate *P* < 0.05, which was considered statistically significant.

ACN, acinar; ade, adenocarcinoma; ARMS, amplification-refractory mutation system; LCNEC, large-cell neuroendocrine carcinoma of the lung; LEP, lepidic; MIP, micropapillary; NGS, next-generation sequencing; PAP, papillary; PD-L1, programmed death ligand-1; SARC, sarcoma; SOL, solid; TMB, tumor mutational burden.

^a^Age was classified as old or young according to the mean age of 61 years.

^b^The subtypes of adenocarcinoma.

^c^*P*-value was calculated using the Mann–Whitney U test.

To evaluate potential assay-related bias, sensitivity analyses were performed in the AdjGroup restricted to patients assessed exclusively with the 22C3 assay.

### Next-generation Sequencing and Genomic Analysis

Tumor DNA was extracted from FFPE samples using the QIAamp DNA FFPE Tissue Kit (Qiagen, Hilden, Germany). Samples with an estimated tumor cell content >20% were considered adequate for genomic analysis. DNA quality and quantity were assessed using NanoDrop and Qubit assays.

For the 520-gene panel (OncoScreen Plus; Burning Rock Biotech, Guangzhou, China), DNA fragments (200–400 bp) were purified using an Agencourt AMPure XP Kit (Beckman Coulter, CA, USA), followed by end repair, adaptor ligation, polymerase chain reaction amplification, and hybridization-based target capture enrichment. The final library was analyzed for fragment size distribution and molar concentration using the Agilent 2100 Bioanalyzer system with the High Sensitivity DNA kit (Agilent Technologies). Indexed libraries were sequenced on an Illumina NextSeq 500 platform with paired-end reads, achieving a mean sequencing depth of approximately 1000× for tissue samples.

For the 425-gene panel (Geneseeq Prime; Geneseeq Technology Inc., Nanjing, China), genomic DNA was fragmented to 300–350 bp. Libraries were prepared using the KAPA Hyper Prep kit (KAPA Biosystems), followed by adaptor ligation, polymerase chain reaction amplification, and hybridization-based enrichment using customized capture probes. Sequencing was performed on an Illumina HiSeq 4000 platform with an average effective depth of approximately 500× after duplicate removal.

Sequence data were aligned to the human reference genome (hg19) using the Burrows-Wheeler Aligner. Local realignment, duplicate marking, and variant calling were performed using the Genome Analysis Toolkit (GATK) and VarScan. Somatic variants were filtered to exclude germline polymorphisms using population databases (ExAC, 1000 Genomes, dbSNP, ESP) and matched normal controls when available. Functional annotation was performed using ANNOVAR and SnpEff. Structural variants and gene fusions were detected using FACTERA, and copy number variations were determined based on normalized depth-of-coverage analysis relative to reference controls.

The TMB of each tumor was calculated as the number of non-synonymous somatic mutations divided by the total coding region size of the panel used. We counted the non-synonymous single nucleotide variants and indels (within the coding region and splice sites ± 2 bp) with an allelic fraction ≥2% in tissue samples (≥0.2% for plasma samples), excluding hotspot mutations, copy number variations, structure variations, and germline single nucleotide polymorphisms. The size of the coding region was 1.003 and 1.28 Mb for the 520-gene and 425-gene panels, respectively.

Somatic mutation analysis was performed using the maftools R package (v2.18.0).^34^ Mutation annotation format files derived from targeted sequencing were used to generate oncoplots summarizing mutation types, frequencies, and co-occurrence patterns across PD-L1 TPS subgroups. Genes were categorized into canonical oncogenic signaling pathways (RTK–RAS, PI3K, TP53, Hippo, WNT, NOTCH, TGF-β, Cell cycle, NRF2, and MYC).^34^ The fraction of pathway alterations and affected samples was computed descriptively using maftools pathway analysis functions.

Differences in mutation frequency between PD-L1 TPS groups were evaluated using two-sided Fisher’s exact tests. *P* values were visualized in volcano plots generated using R software (RStudio v4.2.0).

Survival analyses in external validation cohorts were conducted using the Kaplan–Meier method, with differences compared by the log-rank test. Hazard ratios (HRs) and 95% confidence intervals (CIs) were estimated using Cox proportional hazards regression models. A two-sided *P*-value <0.05 was considered statistically significant.

### Statistical Analysis

Statistical analyses were performed using R software (RStudio v4.2.0). The cut-off date for follow-up was October 31, 2024. Categorical variables were analyzed using the chi-squared test or Fisher's exact test. Continuous variables were compared using the t-test (if normally distributed) or the Mann–Whitney U/Wilcoxon U test (if non-parametric). Paired continuous variables were compared using the Wilcoxon signed-rank test.

DFS was defined as the duration from surgery until tumor progression, recurrence (based on pathological or radiological evidence), or death from any cause, whichever occurred first. OS was defined as the time from surgery to death from any cause or the last follow-up. DFS and OS were estimated using the Kaplan–Meier method and compared using the log-rank test. Cox proportional hazards regression models were used to evaluate the relative risk of the variables for DFS or OS. Therapeutic heterogeneity was addressed through stratified subgroup analyses according to treatment type.

We explored the correlation between radiological tumor size reduction and pathological tumor regression in NeoGroup using Pearson correlation analysis. The permutation test was used to calculate TPS changes from baseline biopsy to surgical specimens after neoadjuvant immunochemotherapy. A signed logarithmic transformation was applied to normalize the skewed distribution values while preserving the direction of change. The transformation is defined as follows:

$${\mathrm{log}}\_{\text{change }} = {\text{ sign}}\left( {\Delta {\mathrm{TPS}}\% } \right) \, \times {\text{ log }}\left( {{1 } + \, |\Delta {\mathrm{TPS}}\% |} \right)$$

where ΔTPS% represents the percentage of TPS change (ΔTPS% = [TPS of surgical specimen—TPS of baseline biopsy] / TPS of baseline biopsy). All statistical tests were two-tailed, and statistical significance was set at *P* < 0.05.

## Results

### Patient Characteristics

In total, 2269 patients were screened: AdjGroup (N=499) and NeoGroup (*N*=43) (Figure S1A).

**Fig. 1:**
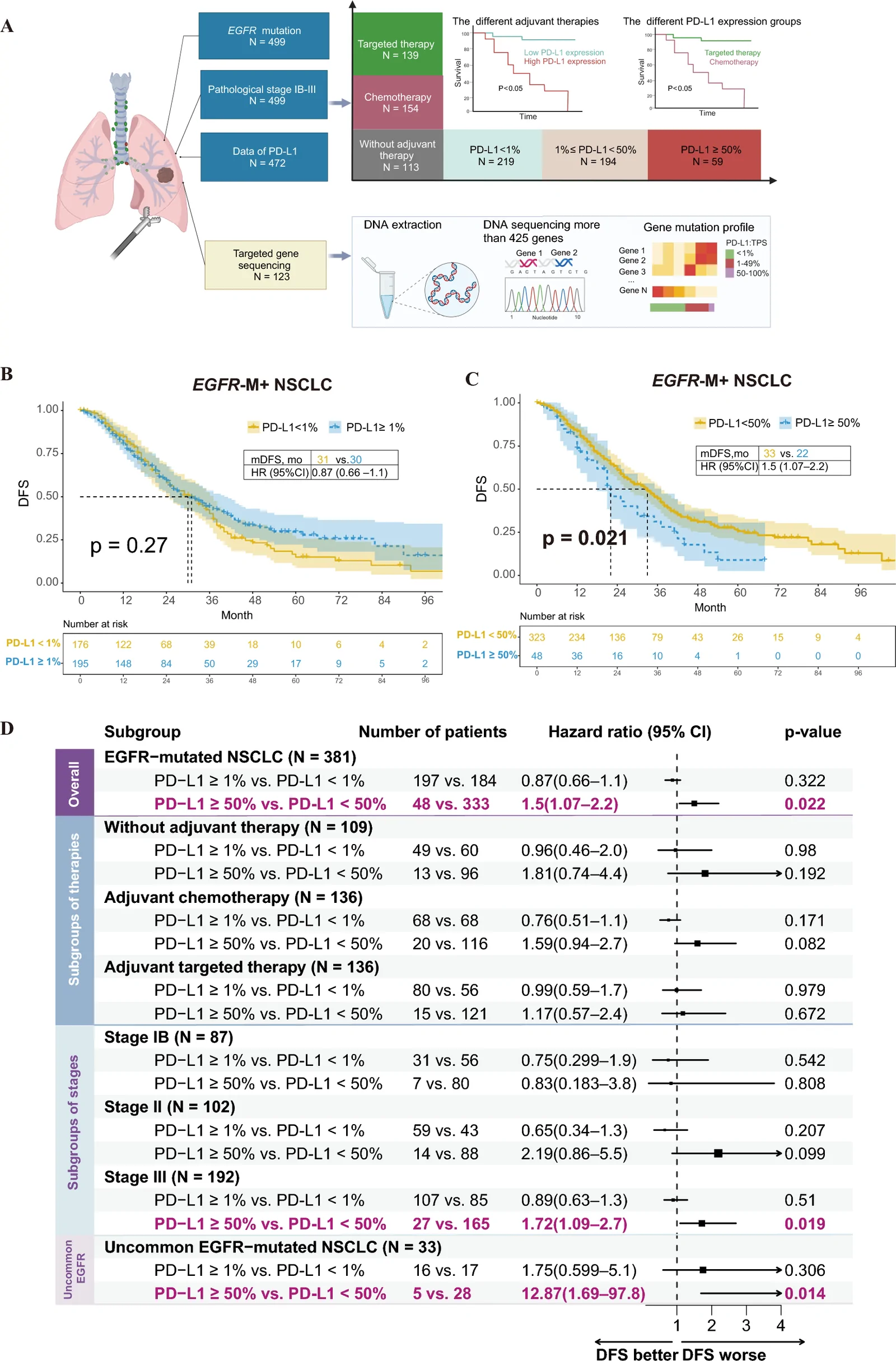
Study design of the AdjGroup and prognostic impact of programmed death ligand-1 (PD-L1) expression in *EGFR-*mutated (*EGFR*-M) non-small-cell lung cancer (NSCLC). (**A**) Patient inclusion criteria for the AdjGroup, with prognostic analyses (N = 472) and next-generation sequencing data (N = 123) stratified according to PD-L1 tumor proportion score (TPS). (**B,C**) Disease-free survival (DFS) for patients in the AdjGroup (N = 472), stratified by PD-L1 TPS (1% and 50%). This cohort includes patients with various adjuvant therapies. (**D**) Subgroup analyses of DFS according to PD-L1 TPS status (cut-offs: 1% or 50%) among AdjGroup patients. Hazard ratios (HRs) and their 95% confidence intervals (CIs) were calculated using multivariate Cox regression models, adjusted for types of adjuvant therapy and other relevant clinical covariates. Additional details of these models are presented in Supplementary Figure S1 and S2. mDFS, modified DFS; mo, month(s)

Table 1 represents patient characteristics with *EGFR*-M+ NSCLC undergoing direct surgery in the AdjGroup (Figure 1A). Overall, pathological stage IIIA was the most common (49.30%), and stage IIA was the least frequent (2.20%). In total, 22.65% of patients did not receive adjuvant therapy, 30.86% received only chemotherapy, 27.86% received only EGFR-TKIs, 5.81% received other therapies, and 12.83% received unknown adjuvant therapy regimens. Further, 44.29% had an exon 19 deletion, 46.89% had an L858R mutation, and 8.82% had uncommon *EGFR* mutations (Table S2). PD-L1 data were available for 472 patients (Table 1). Of these, 46.4% had PD-L1 TPS <1%, 41.1% had TPS 1–49%, and 12.5% had TPS ≥50%.

NeoGroup (*N* = 43) (Table 2) included 31 patients with stage IB–III *EGFR*-M+ NSCLC who received immunochemotherapy and 12 with stage IIIB–IVB *EGFR*-M+ NSCLC who underwent induction/conversion immunochemotherapy before surgery. All NeoGroup patients had an R0 resection. *EGFR* alterations included exon 19 Del in 9 (20.93%), L858R in 10 (23.26%), and exon 20 insertion in six (13.95%) patients. Twelve patients (27.91%) harbored other *EGFR* mutations, including *EGFR*-KDD, G719A, G719S/L861Q, G719X/S768I, V769L, L861Q/R776H, and R831H. The remaining six patients (13.95%) had *EGFR* amplifications. Before surgery, most patients (83.72%) received three cycles of platinum-based chemotherapy combined with ICIs. The median PD-L1 TPS in the baseline biopsies was 2% (interquartile range 0–25) (Table 2).

**Table 2:** Characteristics of patients with *EGFR*-mutated non-small-cell lung cancer receiving immunochemotherapy followed by pulmonary surgery (NeoGroup)

Category	Details	Overall	PD-L1 <1%	1% ≤ PD-L1 <50%	PD-L1 ≥50%	*P*-value
N		43	15 (34.88)	19 (44.19)	9 (20.93)	
Age	Old (≥ 60)	21 (48.84)	8 (53.33)	10 (52.63)	3 (33.33)	0.5779
	Young (< 60)	22 (51.16)	7 (46.67)	9 (47.37)	6 (66.67)	
Sex	Female	22 (51.16)	8 (53.33)	10 (52.63)	4 (44.44)	0.9016
	Male	21 (48.84)	7 (46.67)	9 (47.37)	5 (55.56)	
TNM baseline	IB	2 (4.65)	2 (13.33)	0 (0.00)	0 (0.00)	0.7224
	IIB	8 (18.60)	2 (13.33)	4 (21.05)	2 (22.22)	
	IIIA	21 (48.84)	8 (53.33)	9 (47.37)	4 (44.44)	
	IIIB	7 (16.28)	3 (20.00)	3 (15.79)	1 (11.11)	
	IIIC	1 (2.33)	0 (0.00)	1 (5.26)	0 (0.00)	
	IVA	2 (4.65)	0 (0.00)	1 (5.26)	1 (11.11)	
	IVB	2 (4.65)	0 (0.00)	1 (5.26)	1 (11.11)	
Histology baseline	Adenocarcinoma	31 (72.09)	12 (80.00)	14 (73.68)	5 (55.56)	0.2648
	Adenosquamous	2 (4.65)	0 (0.00)	2 (10.53)	0 (0.00)	
	LELC	1 (2.33)	0 (0.00)	0 (0.00)	1 (11.11)	
	MEC	1 (2.33)	1 (6.67)	0 (0.00)	0 (0.00)	
	Squamous	8 (18.60)	2 (13.33)	3 (15.79)	3 (33.33)	
Biopsy tissue	Lymph node	9 (21.95)	5 (35.71)	4 (22.22)	0 (0.00)	0.13
	Lung	32 (78.05)	9 (64.29)	14 (77.78)	9 (100.00)	
PD-L1% baseline^a^		2 (0–25)	0 (0–0)	5 (2–10)	70 (60–80)	<0.0001
*EGFR* subtype	19Del	9 (20.93)	3 (20.00)	5 (26.32)	1 (11.11)	0.7721
	L858R	10 (23.26)	3 (20.00)	3 (15.79)	4 (44.44)	
	20Ins	6 (13.95)	3 (20.00)	3 (15.79)	0 (0.00)	
	Other	12 (27.91)	4 (26.67)	5 (26.32)	3 (33.33)	
	Amplification	6 (13.95)	2 (13.33)	3 (15.79)	1 (11.11)	
*EGFR* method	ARMS	1 (2.44)	0 (0.00)	1 (5.26)	0 (0.00)	0.5621
	NGS	37 (90.24)	14 (100.00)	16 (84.21)	7 (87.50)	
	NGS_POST^b^	3 (7.32)	0 (0.00)	2 (10.53)	1 (12.50)	
*TP53* mutation	No	8 (24.24)	3 (25.00)	3 (21.43)	2 (28.57)	0.9345
	Yes	25 (75.76)	9 (75.00)	11 (78.57)	5 (71.43)	
Radiological change from baseline		-22.660 ± 21.237	-15.925 ± 26.663	-29.434 ± 17.850	-19.587 ± 14.221	0.164
Pathological tumor regression^a^		84.000 (40.000–99.000)	80.000 (40.000–99.500)	75.000 (35.000–96.750)	90.000 (70.000–99.000)	0.5737
RECIST	PR	19 (44.19)	4 (26.67)	12 (63.16)	3 (33.33)	0.0925
	SD	22 (51.16)	9 (60.00)	7 (36.84)	6 (66.67)	
	PD	2 (4.65)	2 (13.33)	0 (0.00)	0 (0.00)	
Pathologic assessment	pCR	8 (18.60)	3 (20.00)	3 (15.79)	2 (22.22)	0.9033
	0%<RVT≤10%	10 (23.26)	3 (20.00)	4 (21.05)	3 (33.33)	
	non-MPR	25 (58.14)	9 (60.00)	12 (63.16)	4 (44.44)	
Neoadjuvant therapy	2 cycles	5 (11.63)	1 (6.67)	2 (10.53)	2 (22.22)	0.5241
	3 cycles	36 (83.72)	14 (93.33)	16 (84.21)	6 (66.67)	
	4 cycles	2 (4.65)	0 (0.00)	1 (5.26)	1 (11.11)	
Surgery	Pneumonectomy	2 (4.65)	2 (13.33)	0 (0.00)	0 (0.00)	0.2607
	Lobectomy	38 (88.37)	13 (86.67)	16 (84.21)	9 (100.00)	
	Sleeve lobectomy	1 (2.33)	0 (0.00)	1 (5.26)	0 (0.00)	
	Wedge resection	2 (4.65)	0 (0.00)	2 (10.53)	0 (0.00)	
Adjuvant therapy	No	20 (50.00)	7 (50.00)	10 (58.82)	3 (33.33)	0.6709
	Other	7 (17.50)	3 (21.43)	3 (17.65)	1 (11.11)	
	Radiation therapy	2 (5.00)	0 (0.00)	1 (5.88)	1 (11.11)	
	Targeted therapy	11 (27.50)	4 (28.57)	3 (17.65)	4 (44.44)	

Data are presented as n (%), mean ± standard deviation, or median (interquartile range) unless otherwise indicated.

ARMS, amplification-refractory mutation system; LELC, lymphoepithelioma-like carcinoma; MEC, mucoepidermoid carcinoma; MPR, major pathological response; NGS, next-generation sequencing; pCR, pathological complete response; PD, progressive disease; PD-L1, programmed death ligand-1; PR, partial response; RVT, residual viable tumor; SD, stable disease; TNM, tumor, node, metastasis.

^a^*P*-value was calculated with Mann–Whitney U test.

^b^NGS_POST indicates mutations detected in the surgical specimen and that mutation data from baseline biopsies were not available.

### Prognosis Impact of PD-L1 in the AdjGroup

In the AdjGroup, we analyzed the association between PD-L1 expression and survival. We then focused on 381 patients with *EGFR*-M+ NSCLC at pathological stages IB–III in the AdjGroup who received no adjuvant therapy, adjuvant chemotherapy, or adjuvant EGFR-TKIs. The median follow-up period was 46 months; 205 patients experienced tumor recurrence or death. Patients with *EGFR*-M+ NSCLC with TPS ≥50% had significantly shorter DFS. The median DFS was 22 months for the PD-L1 ≥50% group versus 32 months for the TPS <50% group (HR 1.5; 95% CI 1.07–2.2; *P* = 0.022) (Figure 1C and Supplementary Figure S2B). There was no significant difference when using a PD-L1 cut-off of 1% (HR 0.87; 95% CI 0.66–1.1; P = 0.322) (Figure 1B and Supplementary Figure S2A). Sensitivity analyses restricted to patients tested using the 22C3 assay yielded consistent results, with PD-L1 ≥50% remaining significantly associated with shorter DFS (Figure S2G, H).

**Fig. 2:**
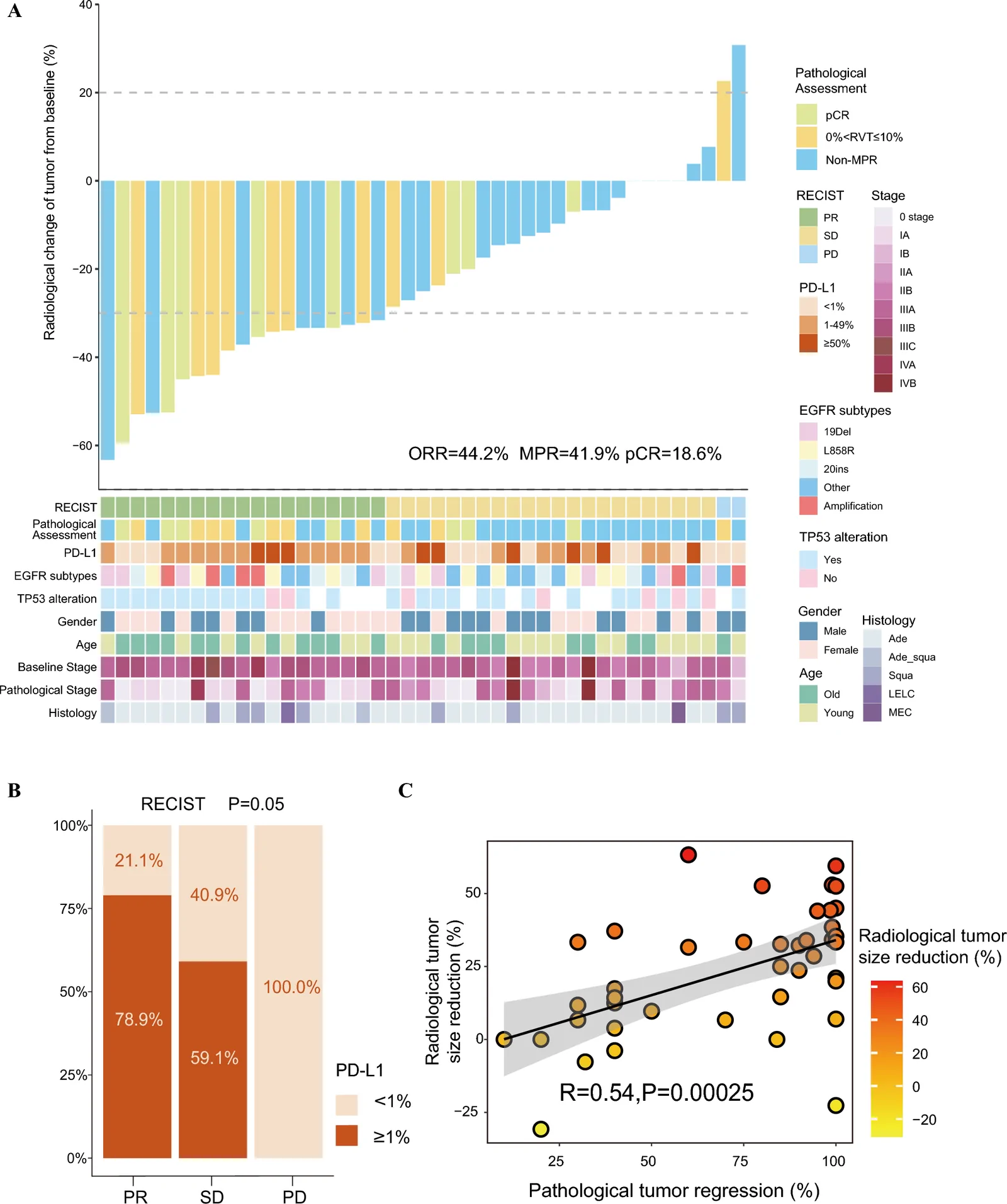
Efficacy of induced immunochemotherapy and its association with baseline programmed death-ligand 1 (PD-L1) expression in *EGFR*-mutated non-small-cell lung cancer (NeoGroup). (**A**) Radiological tumor response in the NeoGroup (N = 43). Pathological assessment was categorized as: pathologic complete response (pCR) (residual viable tumor [RVT] = 0%), major pathologic response (MPR) (RVT ≤10%), or non-MPR (RVT >10%). Color codes: pCR (light green), 0 < RVT ≤ 10% (bright yellow), non-MPR (bright blue). Baseline characteristics are detailed in Table 2. (**B**) Distribution of PD-L1 expression across radiological response categories. Statistical significance was assessed using a two-sided Fisher’s exact test. (**C**) Correlation between pathological tumor regression (%) and radiological tumor size reduction (%). Each dot’s color represents the degree of radiological tumor size reduction. Positive values on the y-axis indicate tumor reduction. Pearson’s correlation analysis was used to assess relationships. Ade, adenocarcinoma; Ade_squa, adenosquamous; LELC, lymphoepithelioma-like carcinoma; MEC, mucoepidermoid carcinoma; ORR, overall response rate; PD, progressive disease; PR, partial response; SD, stable disease; Squa, squamous

Outcomes in specific subgroups were analyzed. Among the 136 patients in the AdjGroup who received adjuvant EGFR-TKIs, PD-L1 status was not associated with prognosis. In this subset, neither PD-L1 ≥50% nor PD-L1 ≥1% was associated with differences in DFS (for PD-L1 ≥1% vs. <1%: HR 0.99; 95% CI 0.59–1.7; for PD-L1 ≥50% vs. <50%, HR 1.17; 95% CI 0.57–2.4; *P* > 0.5) (Figure 2D, Supplementary Figure S1B,C and Supplementary Figure S2C,D).

We also analyzed the prognostic effects of PD-L1 across various AdjGroup subgroups (Figure 1D), which included patients stratified by the adjuvant therapy type (none, chemotherapy, TKIs), pathological stage (IB, II, III), and *EGFR* mutation subtype. PD-L1 ≥50% was associated with poor DFS in most subgroups. The adverse effect of high PD-L1 expression was most significant in patients with pathological stage III disease and in those with uncommon *EGFR* mutations.

### Predictive Role of PD-L1 in NeoGroup

The median follow-up time for the NeoGroup was 11.3 months. At the data cut-off point, the median DFS was 22.9 months (95% CI 15.0–not achieved) (Supplementary Figure S3A). Radiological tumor response was measured using the Response Evaluation Criteria in Solid Tumors (RECIST), version 1.1.^35,36^ The objective response, stable disease (SD), and progressive disease (PD) rates were 44.19%, 51.16%, and 4.65%, respectively (Figure 2A). Regarding pathological assessment, 18 patients (41.9%) achieved a major pathologic response (MPR), including eight patients (18.6%) with a pathologic complete response (pCR) (Figure 2A).

We analyzed whether baseline PD-L1 expression could predict tumor response to immunochemotherapy in the NeoGroup. All patients with tumor PD had a PD-L1 TPS <1% at baseline (Figure 2B). Patients with tumor partial responses (PRs) had a high prevalence of positive PD-L1 expression. The proportion of patients with PD-L1 TPS ≥1% at baseline was 78.9% in the PR group, 59.1% in the SD group, and 0% in the PD group (*P* = 0.05) (Figure 2B), demonstrating the association between baseline PD-L1 expression and radiological tumor shrinkage during immunochemotherapy (Supplementary Figure S3B).

Pathological tumor regression was positively correlated with radiological tumor size reduction (*R* = 0.54, *P* = 0.00025) (Figure 2C). Moreover, *TP53* mutations were potentially related to tumor response. All patients with pCR had *TP53* co-mutation, compared with 75.0% among patients with 0% < residual viable tumor (RVT) ≤ 10% and 66.7% among those with non-MPR, with no significant difference (*P* = 0.312) (Supplementary Figure S3E).

### Dynamic Changes in PD-L1 Expression in the NeoGroup

**Fig. 3:**
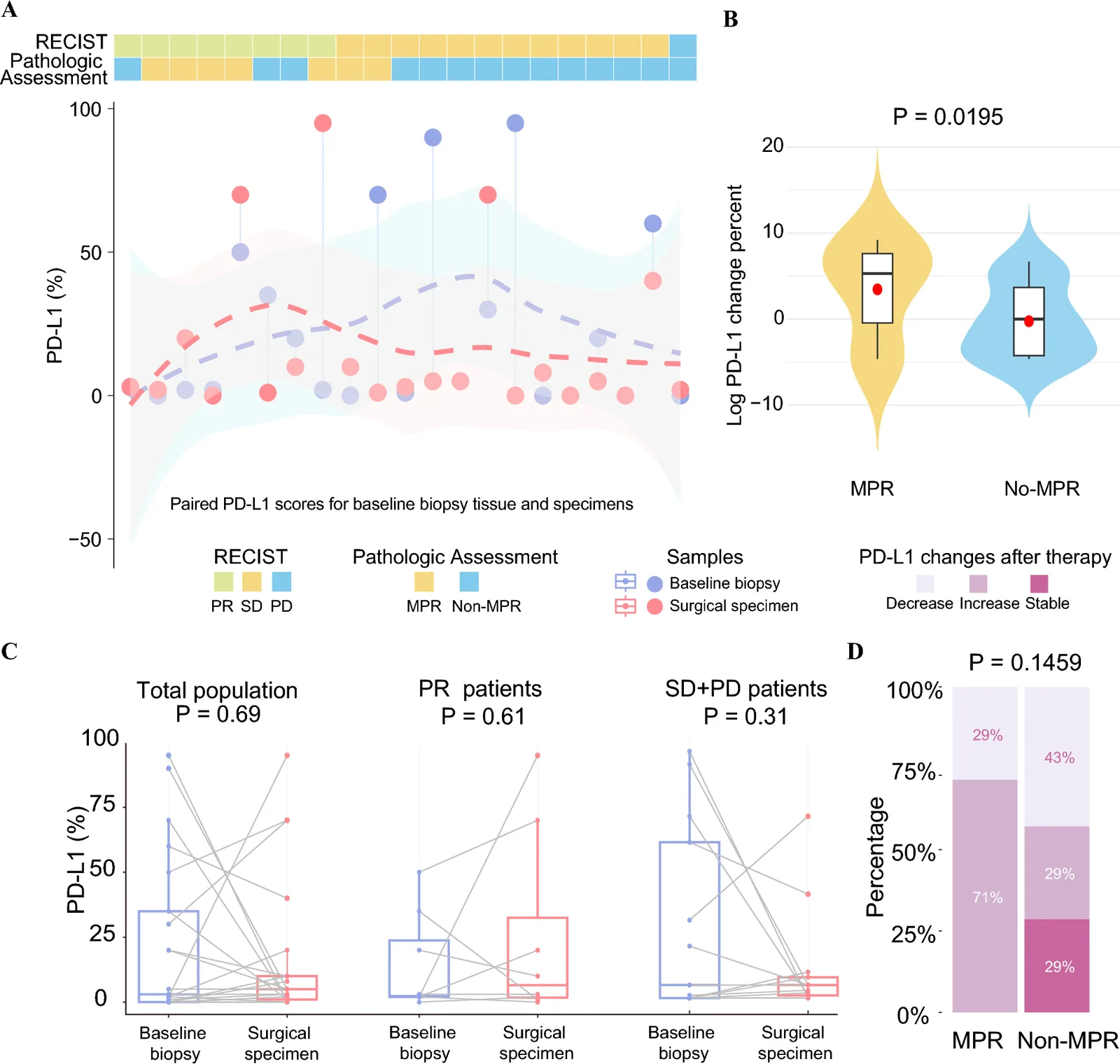
Dynamic changes in programmed death ligand-1 (PD-L1) expression after immunochemotherapy in *EGFR*-mutated non-small-cell lung cancer (NeoGroup). (**A**) Paired comparisons of PD-L1 tumor proportion score (TPS) between baseline biopsies and corresponding surgical specimens (N = 21). Baseline samples obtained from lymph nodes and surgical specimens achieving pathological complete response were excluded. (**B**) Comparison of dynamic PD-L1 changes between patients experiencing a major pathological response (MPR) and those not (non-MPR). PD-L1 change percentages were log-transformed for visualization purposes; statistical analyses were conducted on original values using a permutation test. Red dots indicate the mean values of the y-axis. (**C**) Paired analysis of PD-L1 TPS changes in NeoGroup patients (N = 21), stratified by radiological response: partial response (PR; N = 8) and stable disease + progressive disease (SD+PD; N = 13). Statistical significance was assessed using a two-sided paired Wilcoxon signed-rank test. (**D**) Frequency distribution of PD-L1 change patterns in MPR (N = 7) versus non-MPR patients (N = 14). Statistical significance was determined using a two-sided Fisher’s exact test. The overall distribution of PD-L1 TPS changes for all patients is presented in Supplementary Figure S2C and Figure S2D

Dynamic changes in PD-L1 TPS from baseline biopsy to surgical specimens before and after immunochemotherapy were assessed (Figure 3). Paired PD-L1 data from lungs were available for 21 patients. Missing paired data were mainly due to the absence of residual tumor in patients achieving pCR, insufficient tumor material for PD-L1 assessment, or differences in sampling sites between baseline and surgical specimens. The PD-L1 TPS increased in 42.9% of patients, decreased in 38.1%, and remained unchanged in 19.0% (Supplementary Figure S3C,D).

Patients were evaluated using radiological tumor assessment (Figure 3A). Overall, patients who achieved PR tended to have an increase in PD-L1 TPS after immunochemotherapy. In contrast, some patients with SD or PD showed a decrease in PD-L1 TPS (Figure 3C). These trends were insignificant because of the high variability.

When analyzing pathological response, patients who had an MPR had a greater increase in PD-L1 TPS from baseline (*P* = 0.0195) (Figure 3B). Approximately 71% of patients with MPR had an increase in PD-L1 TPS after therapy, compared with only 29% of patients with non-MPR (*P* = 0.1459) (Figure 3D).

Overall, our data demonstrated PD-L1 upregulation in tumors with favorable responses to immunochemotherapy in *EGFR*-M+ NSCLC.

### PD-L1 Expression and Clinicopathological Features in the AdjGroup

**Fig. 4:**
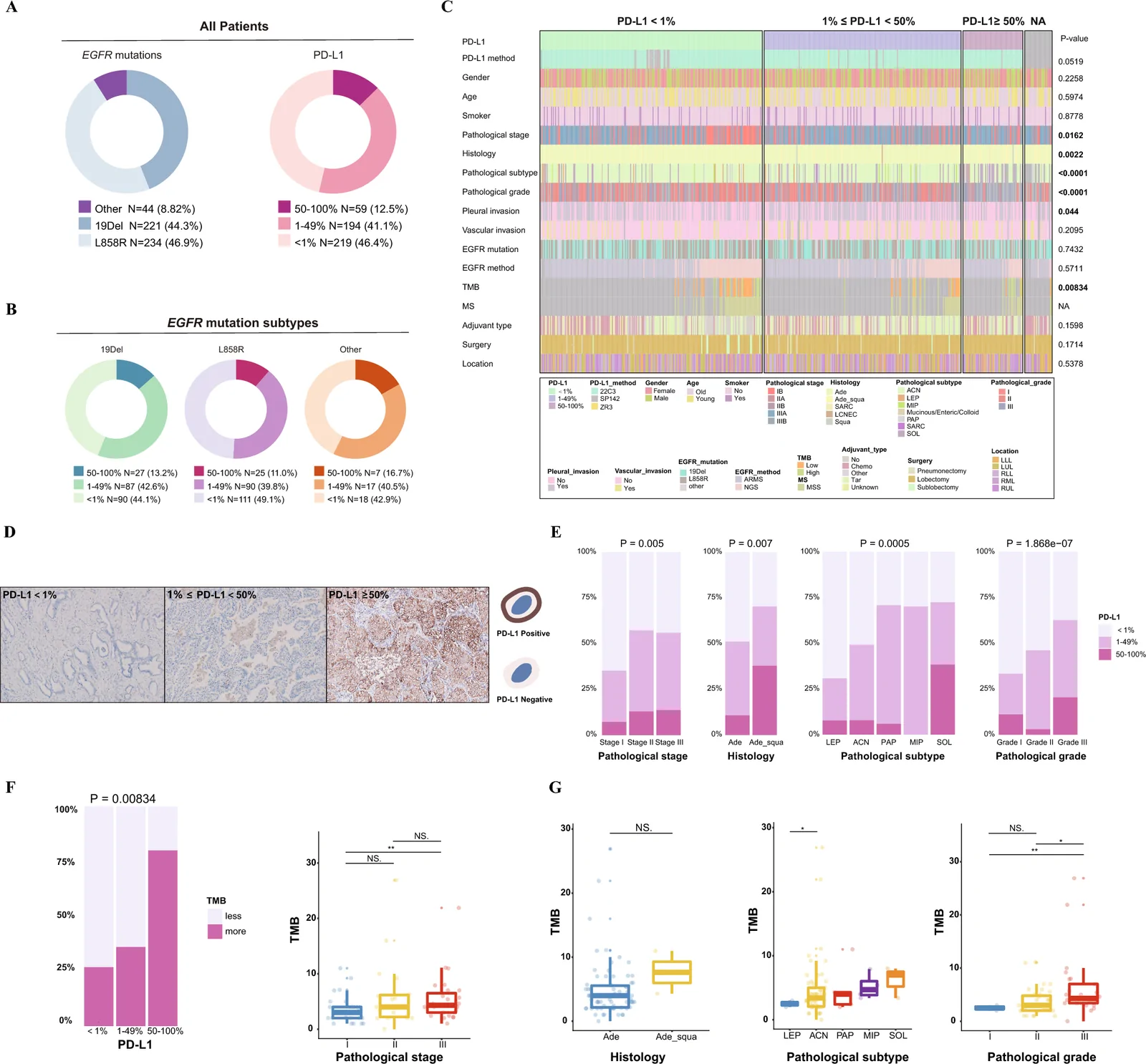
Clinicopathological characteristics of programmed death ligand-1 (PD-L1) expression and tumor mutational burden (TMB) in patients with *EGFR*-mutated disease (AdjGroup). (**A**) Distribution of *EGFR* mutation subtypes (left panel, N = 499) and corresponding PD-L1 tumor proportion score (TPS) categories (right panel, N = 472). (**B**) PD-L1 TPS distribution across different *EGFR* mutation subtypes. (**C**) Clinicopathological characteristics of patients with *EGFR*-M+ non-small-cell lung cancer stratified by PD-L1 TPS groups (N = 499). Age is stratified by the average value of 60 years as old and young. TMB is classified as low or high based on a mean cut-off value of 4.6 mutations/Mb (details provided in Table 1). (**D**) Representative immunohistochemistry images illustrating PD-L1 TPS: TPS <1% (left), 1–49% (middle), and ≥50% (right). (**E**) Distribution of PD-L1 TPS across various pathological characteristics. Statistical significance was evaluated using chi-squared or Fisher's exact tests. More details in Supplementary Figure S4C. (**F,G**) Associations between pathological features, PD-L1 TPS, and TMB. Statistical comparisons between groups were performed using Fisher's exact tests and the Wilcoxon rank-sum test. *P < 0.05, **P < 0.01, ***P < 0.001. Ade, adenocarcinoma; Ade_squa, adenosquamous; ACN, acinar; LEP, lepidic; MIP, micropapillary; NS, not significant; PAP, papillary; SOL, solid

Clinicopathological characteristics of PD-L1 expression in the AdjGroup (N = 499) are represented in Figure 4A and Table 1. No significant differences in PD-L1 expression were observed among the three *EGFR* mutation subtypes (*P* = 0.7432) (Figure 4B).

Pathological stage (*P* = 0.0162), histology (P = 0.0022), pathological subtype (*P* < 0.0001), pathological grade (*P* < 0.0001), and pleural invasion (*P* = 0.044) (Figure 4C) were significantly associated with PD-L1 TPS. Representative PD-L1 immunohistochemically stained slides of NSCLC are shown in Figure 4D. The PD-L1 TPS tended to be higher in patients with *EGFR*-M+ NSCLC with an advanced pathological stage, mixed histology, aggressive pathological subtype, and higher pathological grade (Figure 4E) (Supplementary Figure S4C).

The median TMB for this subset (N = 78) was 3.995 mutations/Mb. There was a significant difference in the TMB values across the PD-L1 groups (P = 0.0067) (Table 1). Tumors with PD-L1 ≥50% had higher median TMB than those with PD-L1 <1%. When TMB was separated into high versus low using the mean of 4.6, the proportion of high-TMB cases was highest in the PD-L1 ≥50% group (P = 0.00834) (Figure 4F). The TMB value is also related to pathological stage, subtype, and grade.

### Genomic Correlates of PD-L1 Expression in the AdjGroup

**Fig. 5:**
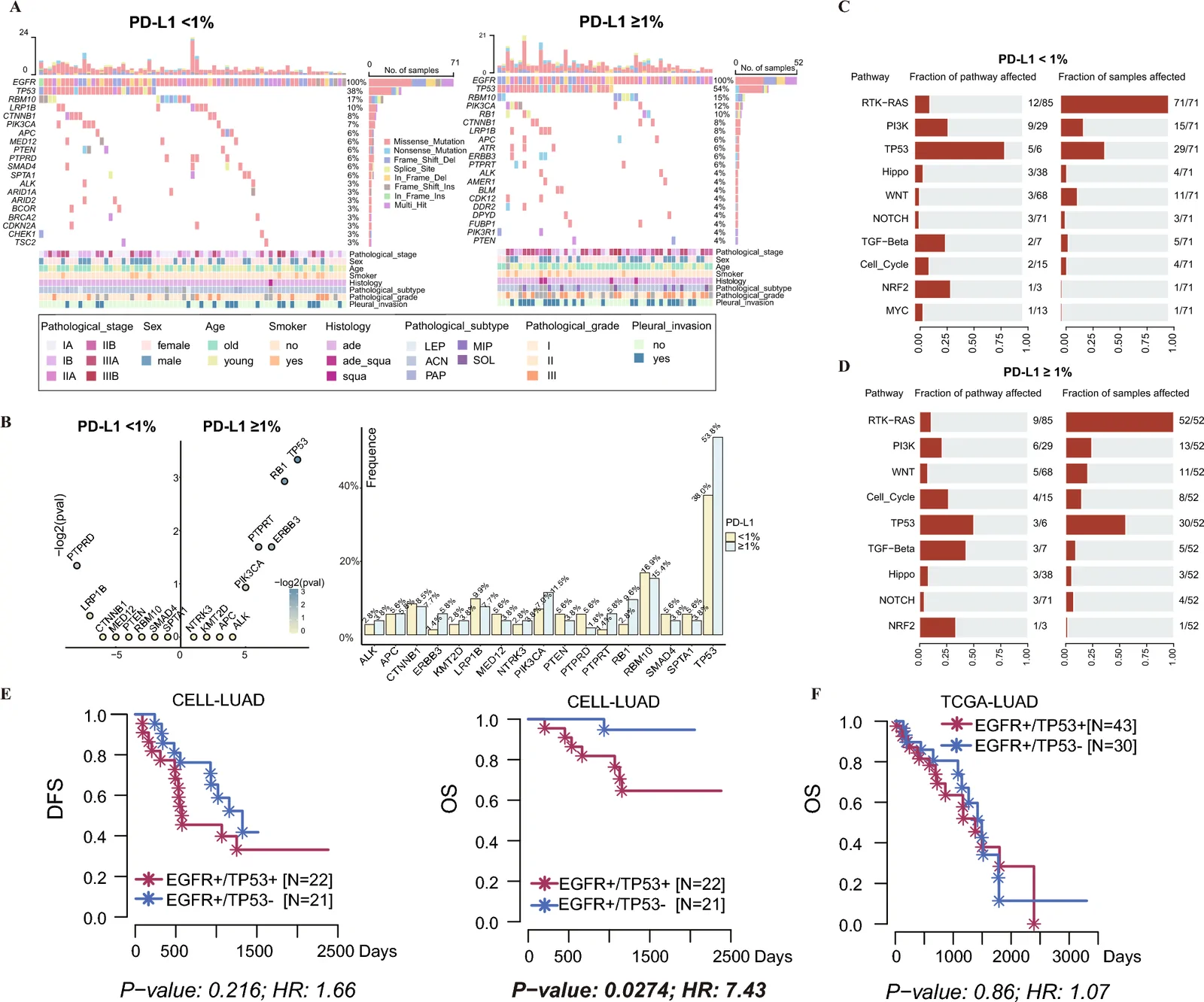
Genomic alterations stratified by programmed death ligand-1 (PD-L1) expression and prognostic impact of *TP53* co-mutation in *EGFR*-mutated non-small-cell lung cancer (NSCLC) (AdjGroup). (**A**) Genomic mutation landscape and associated clinical features in 123 patients with *EGFR*-M+ NSCLC, stratified by PD-L1 tumor proportion score (TPS) (<1% vs. ≥1%). Additional details are provided in Supplementary Figure S4. (**B**) Comparison of mutation frequencies between PD-L1 <1% and ≥1% groups (N=123). Genes mutated in more than four patients were included. (**C, D**) Signal pathway enrichment analyses for PD-L1 <1% and ≥1% groups (N=123). (**E, F**) Survival outcomes in patients with *EGFR*+/*TP53*± mutated NSCLC in external databases: independent cohort of patients with stage I–III *EGFR*-M+ lung adenocarcinoma (CELL-LUAD; N=43) and patients with *EGFR*-M+ lung adenocarcinoma from The Cancer Genome Atlas (TCGA-LUAD; N=73). Ade, adenocarcinoma; Ade_squa, adenosquamous; DFS, disease-free survival; HR, hazard ratio

To further investigate the gene features related to PD-L1 TPS in *EGFR*-M+ NSCLC, NGS data from 123 patients were analyzed (87 patients from the AdjGroup and an additional 36 patients with stage I disease from the GDPH), with the cut-off of PD-L1 = 1 (TPS ≥1%, N = 71; TPS <1%, *N* = 52) (Figure 5A, Supplementary Figure S4). We compared the frequencies of mutations in genes and pathways.

The most common co-mutations occurring with *EGFR* were *TP53* (45% of patients), *RBM10* (16%), *LRP1B* (9%), *PIK3CA* (9%), and *CTNNB1* (8%) (Supplementary Figure S4). *TP53* mutations were more frequent in patients with PD-L1 ≥1% NSCLC (53.8%) than in those with PD-L1 <1% (38.0%) (Figure 5B). Similarly, *RB1* (9.6% vs. 2.8%), *PTPRT* (5.8% vs. 1.4%), *ERBB3* (5.8% vs. 1.4%), and *PIK3CA* (11.5% vs. 7.0%) mutations were observed at higher rates in PD-L1-positive NSCLC than in PD-L1-negative NSCLC. Thus, PD-L1 expression appeared to be associated with the presence of certain co-mutations, although the differences were statistically insignificant.

As for signaling pathways, 57.69% of NSCLC with PD-L1 ≥1% had alterations in the *TP53* pathway, compared with 40.85% of PD-L1-negative NSCLC (*P* = 0.07) (Figure 5C,D).

We investigated whether *TP53* co-mutations affected the survival outcomes of patients with *EGFR*-M+ NSCLC (Figure 5E, F). In the CELL-LUAD cohort, patients with *EGFR*-M+ NSCLC with *TP53* mutations had a significantly shorter OS (HR 7.43, *P* = 0.0274) and a trend towards shorter DFS (HR 1.66, *P* = 0.216) than patients with *TP53*-wildtype *EGFR*-M+ NSCLC (Figure 5E). Survival outcomes from the TCGA-LUAD cohort also showed a trend towards poorer OS in patients with *EGFR*-M+ NSCLC with *TP53* mutations, although this difference was statistically insignificant (OS: HR 1.07, *P* = 0.86).^31^ These external analyses demonstrated that *TP53* mutations, which frequently coexist with PD-L1 expression, may correlate with poorer prognosis in *EGFR*-M+ NSCLC. This was consistent with our finding from the AdjGroup that patients with PD-L1-positive *EGFR*-M+ NSCLC, who often harbor *TP53* mutations, had poorer survival without immunotherapy.

## Discussion

For patients with *EGFR*-M+ NSCLC, EGFR-TKIs are associated with improved survival outcomes. However, tumor recurrence rates within 2–3 years remain substantial.^3,5,37,38^ ICIs have shown remarkable efficacy in *EGFR*-wildtype NSCLC, particularly in patients with high PD-L1 expression. The use of ICIs in the early and locally advanced stages of *EGFR*-M+ NSCLC has been limited, and their benefits remain unclear, as patients with *EGFR*-M+ NSCLC were often excluded from relevant clinical trials.^9,39–43^ Our study highlights the necessity of closely monitoring patients with *EGFR*-M+ NSCLC with high PD-L1 expression, as they may face a higher risk of early recurrence under standard treatment in real-world settings. Our findings also suggest that the addition of immunotherapy to the standard treatment for such cases during the perioperative period may improve survival outcomes.

### PD-L1 as a Prognostic Biomarker in Resectable *EGFR*-M+ NSCLC

In the AdjGroup, PD-L1 ≥50% independently predicted poorer DFS in pathological stage IB–III *EGFR*-M+ NSCLC receiving pulmonary surgery, after adjusting for confounding factors across various adjuvant therapies and clinical features. Patients with PD-L1 ≥50% had a shorter DFS than those with PD-L1 <50% (33 vs. 22 months).

PD-L1 ≥1% was reported to be associated with worse DFS and OS in a cohort of 267 patients with resected *EGFR*-M+ NSCLC.^19^ Unlike previous studies, our cohort was more representative of the adjuvant setting; approximately 19% of patients were stage IB and 49.3% were stage IIIA. However, we could not distinguish OS when grouped by PD-L1 expression possibly due to insufficient follow-up time, differences in PD-L1 clones, intratumoral heterogeneity of PD-L1, and different secondary therapies.^26,28^

### PD-L1 as a predictor of immunochemotherapy benefit in *EGFR*-M+ NSCLC

PD-L1 was revealed to be a predictive biomarker for ICIs, even in *EGFR*-M+ NSCLC. Baseline PD-L1 ≥1% was associated with markedly better radiologic responses to immunochemotherapy. For patients achieving PR, 75% had PD-L1 ≥1%, but none of the patients who achieved PD had PD-L1 ≥1%.

The KEYNOTE-091 study indicated a DFS benefit of adjuvant immunotherapy in the subgroup of *EGFR*-M+ NSCLC compared with adjuvant placebo after chemotherapy (HR 0.44; 95% CI 0.23–0.84).^44^ This indicates the potential benefits of ICIs in perioperative *EGFR*-M+ NSCLC treatment. Additionally, our group’s previous work and other retrospective studies have reported the efficacy of ICIs in patients with resectable NSCLC with positive driver mutations.^25^ Zhang et al. showed that the induction of ICI-related therapies achieved an MPR rate of 37.5% in patients with resectable NSCLC with various driver mutations (*EGFR*, *KRAS, RET,* etc.).^25^ However, their study did not separate the outcomes of patients with *EGFR*-M+ NSCLC because of its small sample size. Our current study focused on *EGFR*-M+ NSCLC and highlighted that PD-L1 expression is related to ICI efficacy. Pang et al.^26^ recently reported that, in patients with advanced *EGFR*-M+ NSCLC who had progressed on TKIs, those with PD-L1 ≥50% had significantly greater benefit from a combination of anti-PD-1/PD-L1 and anti-VEGF therapy (plus chemotherapy) compared with chemotherapy alone, whereas patients with PD-L1 <50% had less benefit (HR 0.24 for PD-L1 ≥50% vs. HR 0.63 for PD-L1 <50%). These results support the idea that high PD-L1 expression is related to the efficacy of ICIs even in *EGFR*-M+ NSCLC populations.^26^

### Dynamic PD-L1 changes and immunochemotherapy response

ICIs can be used to remodel the NSCLC microenvironment. However, the limited use of ICIs in perioperative patients with *EGFR*-M+ NSCLC has restricted our understanding of these changes. We observed that tumors with MPR often showed greater increases in PD-L1 TPS from baseline to post-treatment surgical specimens. This finding raises questions regarding the tumor microenvironment and adaptive immune resistance. ICIs combined with chemotherapy may lead to the upregulation of PD-L1 as a feedback mechanism in *EGFR*-M+ NSCLC. These findings corroborate those of Isomoto et al.,^29^ who reported that subsequent ICI therapy led to prolonged PFS in patients with *EGFR*-M+ NSCLC whose PD-L1 TPS converted from low to high after EGFR-TKI therapy. An increase in the PD-L1 TPS might signify that the tumor may be more “visible” to the immune system and thus more susceptible to ICIs.^26^ Sampling heterogeneity may still influence PD-L1 scores. Therefore, dynamic TPS changes should be interpreted with caution.

### Limitations and Future Directions

This study has several limitations. First, given the retrospective design and the relatively small sample size of the NeoGroup, some exploratory analyses (e.g., the association between *TP53* co-mutations and pathological response) may be underpowered and should be interpreted cautiously.

Second, in the AdjGroup, *TP53* co-mutations were associated with PD-L1 expression and adverse clinical outcomes. Previous clinical evidence has shown that *TP53* alterations are linked to inferior survival and therapeutic resistance in *EGFR*-M+ NSCLC treated with EGFR-TKIs.^45^ Beyond their prognostic role, *TP53* dysfunction has been implicated in promoting chromosomal instability and genome doubling, which can drive intratumoral heterogeneity and diverse evolutionary trajectories under treatment pressure.^46^ Such genomic instability may contribute to increased tumor adaptability and resistance, possibly leading to poorer outcomes in patients harboring *TP53* co-mutations in our cohort and external validation cohorts.

Mechanistic studies suggest potential direct links between *TP53* and PD-L1 regulation (e.g., a p53/miR-34/PD-L1 axis).^47^ However, because our targeted sequencing panels were not designed to comprehensively interrogate immune-regulatory signaling or transcriptomic immune programs, the molecular mechanisms linking *TP53* alterations and PD-L1 expression in *EGFR*-M+ NSCLC remain incompletely defined and warrant further mechanistic and multiomics investigations.

Third, the external validation cohorts differ from our institutional cohort in treatment background and patient selection. Therefore, these datasets were used to confirm the biological association between *TP53* co-mutation and prognosis rather than to reproduce identical clinical outcomes. The consistent trend observed across independent *EGFR*-M+ cohorts supports the robustness of this finding, although some heterogeneity cannot be completely excluded. In addition, real-world treatment heterogeneity may influence outcome interpretation. While stratified analyses were performed to mitigate confounding, residual heterogeneity remains.

Finally, the limited size of the NeoGroup meant that some uncommon *EGFR* alterations were included. Future large-scale prospective studies are warranted to better define the relationships among *EGFR* subtype, PD-L1 expression, and perioperative treatment efficacy.

Despite these limitations, our findings generate testable hypotheses and provide insights for future research. Ongoing prospective trials, including NEOVADE (NCT06300424) and NEOTIDE (CTONG2104), are evaluating neoadjuvant immunochemotherapy-based strategies in *EGFR*-M+ NSCLC and may further inform perioperative patient selection and treatment optimization.^24^

## Conclusions

In *EGFR*-M+ NSCLC, PD-L1 exhibits a dual prognostic and predictive role. In patients treated with surgery and adjuvant therapy, high PD-L1 expression is associated with a higher risk of recurrence and poorer prognosis. However, in patients receiving immunochemotherapy, a higher percentage of baseline-positive PD-L1 expression and a greater increase in PD-L1 TPS after therapy predicted more favorable tumor responses. These findings suggest that PD-L1 status could be used to select perioperative treatment strategies for *EGFR*-M+ NSCLC, indicating which patients may benefit from the addition of immunochemotherapy. Further prospective studies are warranted to validate these observations and improve the outcomes for patients with *EGFR*-M+ NSCLC.

## Supplementary Information

Below is the link to the electronic supplementary material.

Supplementary file1 (PDF 592 KB)

Supplementary file2 (PDF 734 KB)

Supplementary file3 (PDF 586 KB)

Supplementary file4 (PDF 3844 KB)

Supplementary file5 (XLSX 11 KB)

Supplementary file6 (XLSX 12 KB)

## Data Availability

The data generated and analyzed in this study were obtained from clinical diagnostic specimens and are subject to institutional and ethical restrictions. Processed genomic data supporting the findings of this study are available from the corresponding author upon reasonable request for academic purposes and with approval from the institutional ethics committee. The external cohort CELL-LUAD dataset generated during the current study is available from https://doi.org/10.1016/j.cell.2020.05.043.31

## Generative AI and AI-assisted Technologies in the Writing Process

During the preparation of this work, the authors used ChatGPT (OpenAI) solely for language and grammar refinement. After using this tool, the authors reviewed and edited the content as needed and take full responsibility for the content of the publication.
