# Supplementary material for: The Role of PD-L1 in Treatment Decision-Making for Perioperative EGFR-Mutated Lung Cancer and its Genomic Analysis
Source: Ann Surg Oncol. 2026 May 27;33(10):8874–90. doi: 10.1245/s10434-026-19781-0 (PMC13586022; doi:10.1245/s10434-026-19781-0)
Supplement: Supplementary file 1 [file 10434_2026_19781_MOESM1_ESM.pdf]

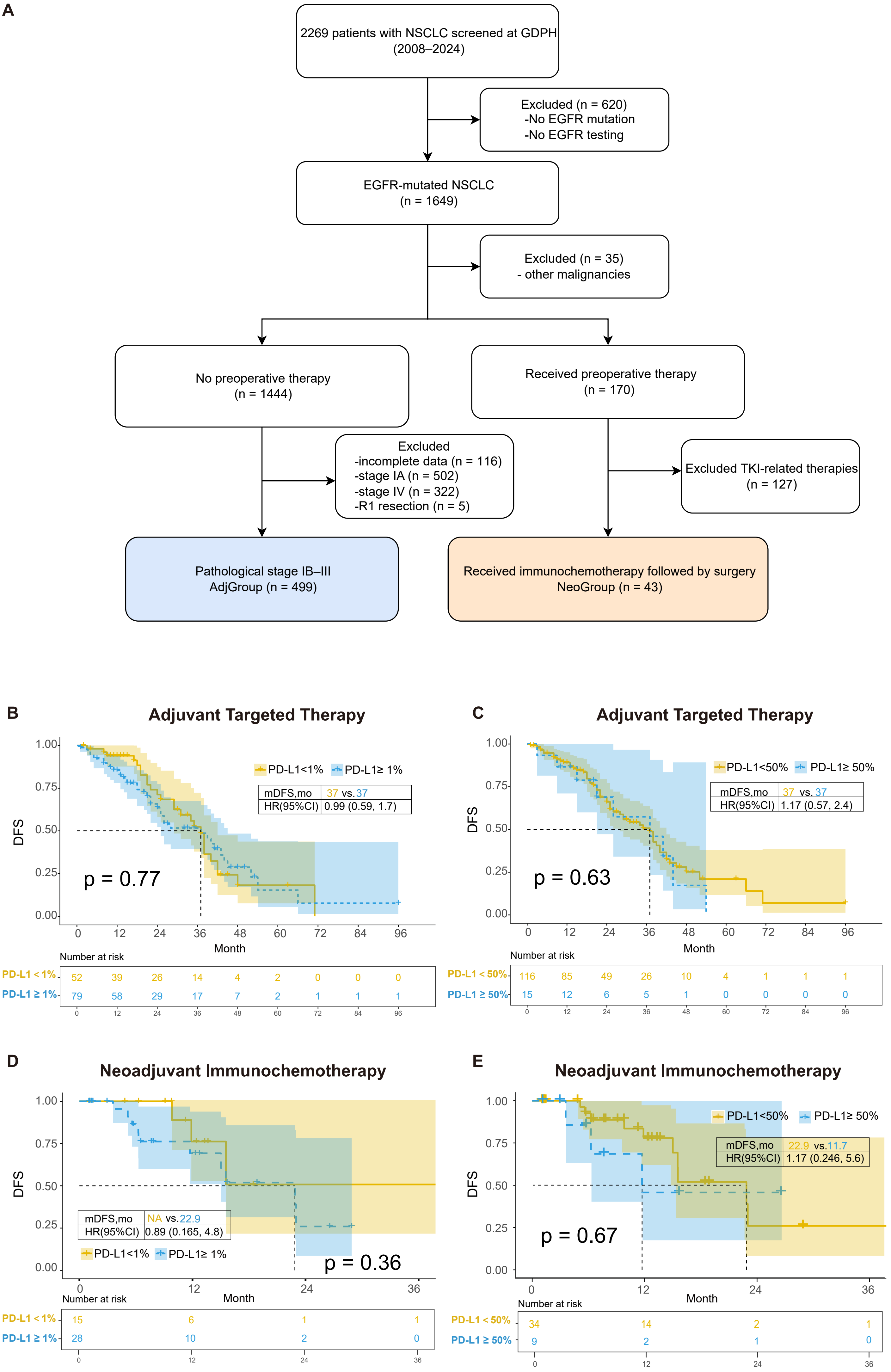

A
2269 patients with NSCLC screened at GDPH (2008–2024)
Excluded (n = 620)
-No EGFR mutation
-No EGFR testing
EGFR-mutated NSCLC (n = 1649)
Excluded (n = 35)
- other malignancies
No preoperative therapy (n = 1444)
Received preoperative therapy (n = 170)
Excluded
-incomplete data (n = 116)
-stage IA (n = 502)
-stage IV (n = 322)
-R1 resection (n = 5)
Excluded TKI-related therapies (n = 127)
Pathological stage IB–III AdjGroup (n = 499)
Received immunochemotherapy followed by surgery NeoGroup (n = 43)
B
Adjuvant Targeted Therapy
PD-L1<1%
PD-L1≥ 1%
mDFS,mo 37 vs. 37
HR(95%CI) 0.99 (0.59, 1.7)
p = 0.77
DFS
Month
Number at risk
PD-L1 < 1% 52 39 26 14 4 2 0 0 0
PD-L1 ≥ 1% 79 58 29 17 7 2 1 1 1
C
Adjuvant Targeted Therapy
PD-L1<50%
PD-L1≥ 50%
mDFS,mo 37 vs. 37
HR(95%CI) 1.17 (0.57, 2.4)
p = 0.63
DFS
Month
Number at risk
PD-L1 < 50% 116 85 49 26 10 4 1 1 1
PD-L1 ≥ 50% 15 12 6 5 1 0 0 0 0
D
Neoadjuvant Immunochemotherapy
mDFS,mo NA vs. 22.9
HR(95%CI) 0.89 (0.165, 4.8)
PD-L1<1%
PD-L1≥ 1%
p = 0.36
DFS
Month
Number at risk
PD-L1 < 1% 15 6 1 1
PD-L1 ≥ 1% 28 10 2 0
E
Neoadjuvant Immunochemotherapy
PD-L1<50%
PD-L1≥ 50%
mDFS,mo 22.9 vs. 11.7
HR(95%CI) 1.17 (0.246, 5.6)
p = 0.67
DFS
Month
Number at risk
PD-L1 < 50% 34 14 2 1
PD-L1 ≥ 50% 9 2 1 0
