# Supplementary material for: The Role of PD-L1 in Treatment Decision-Making for Perioperative EGFR-Mutated Lung Cancer and its Genomic Analysis
Source: Ann Surg Oncol. 2026 May 27;33(10):8874–90. doi: 10.1245/s10434-026-19781-0 (PMC13586022; doi:10.1245/s10434-026-19781-0)
Supplement: Supplementary file 2 [file 10434_2026_19781_MOESM2_ESM.pdf]

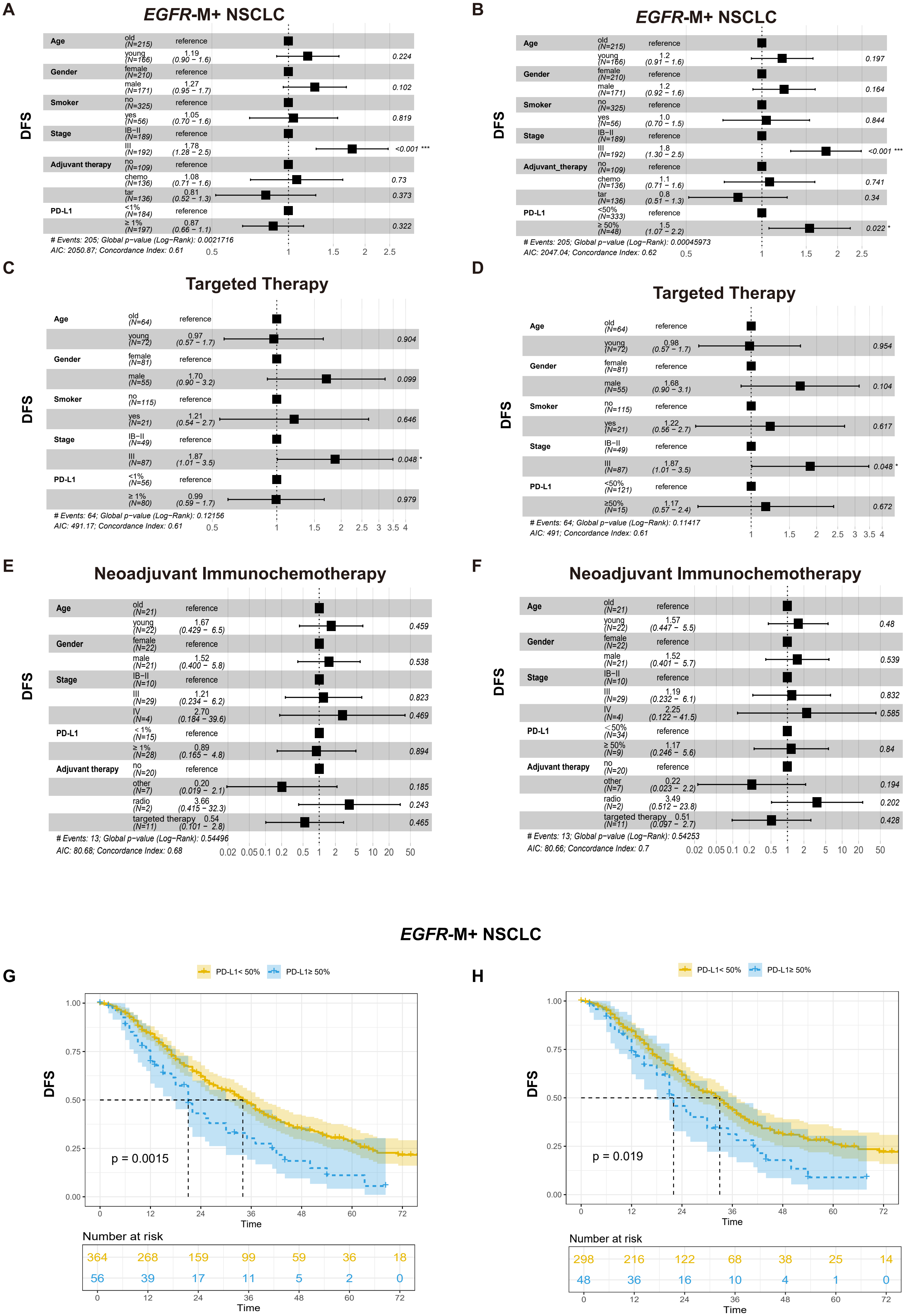

A
EGFR-M+ NSCLC
DFS
Age old (N=215) reference
young (N=166) 1.19 (0.90 − 1.6) 0.224
Gender female (N=210) reference
male (N=171) 1.27 (0.95 − 1.7) 0.102
Smoker no (N=325) reference
yes (N=56) 1.05 (0.70 − 1.6) 0.819
Stage IB−II (N=189) reference
III (N=192) 1.78 (1.28 − 2.5) <0.001 ***
Adjuvant therapy no (N=109) reference
chemo (N=136) 1.08 (0.71 − 1.6) 0.73
tar (N=136) 0.81 (0.52 − 1.3) 0.373
PD-L1 <1% (N=184) reference
≥ 1% (N=197) 0.87 (0.66 − 1.1) 0.322
Events: 205; Global p-value (Log-Rank): 0.0021716
AIC: 2050.87; Concordance Index: 0.61
0.5 1 1.5 2 2.5
B
EGFR-M+ NSCLC
DFS
Age old (N=215) reference
young (N=166) 1.2 (0.91 − 1.6) 0.197
Gender female (N=210) reference
male (N=171) 1.2 (0.92 − 1.6) 0.164
Smoker no (N=325) reference
yes (N=56) 1.0 (0.70 − 1.5) 0.844
Stage IB−II (N=189) reference
III (N=192) 1.8 (1.30 − 2.5) <0.001 ***
Adjuvant_therapy no (N=109) reference
chemo (N=136) 1.1 (0.71 − 1.6) 0.741
tar (N=136) 0.8 (0.51 − 1.3) 0.34
PD-L1 <50% (N=333) reference
≥ 50% (N=48) 1.5 (1.07 − 2.2) 0.022 *
Events: 205; Global p-value (Log-Rank): 0.00045973
AIC: 2047.04; Concordance Index: 0.62
0.5 1 1.5 2 2.5
C
Targeted Therapy
DFS
Age old (N=64) reference
young (N=72) 0.97 (0.57 − 1.7) 0.904
Gender female (N=81) reference
male (N=55) 1.70 (0.90 − 3.2) 0.099
Smoker no (N=115) reference
yes (N=21) 1.21 (0.54 − 2.7) 0.646
Stage IB−II (N=49) reference
III (N=87) 1.87 (1.01 − 3.5) 0.048 *
PD-L1 <1% (N=56) reference
≥ 1% (N=80) 0.99 (0.59 − 1.7) 0.979
Events: 64; Global p-value (Log-Rank): 0.12156
AIC: 491.17; Concordance Index: 0.61
0.5 1 1.5 2 2.5 3 3.5 4
D
Targeted Therapy
DFS
Age old (N=64) reference
young (N=72) 0.98 (0.57 − 1.7) 0.954
Gender female (N=81) reference
male (N=55) 1.68 (0.90 − 3.1) 0.104
Smoker no (N=115) reference
yes (N=21) 1.22 (0.56 − 2.7) 0.617
Stage IB−II (N=49) reference
III (N=87) 1.87 (1.01 − 3.5) 0.048 *
PD-L1 <50% (N=121) reference
≥50% (N=15) 1.17 (0.57 − 2.4) 0.672
Events: 64; Global p-value (Log-Rank): 0.11417
AIC: 491; Concordance Index: 0.61
1 1.5 2 2.5 3 3.5 4
E
Neoadjuvant Immunochemotherapy
DFS
Age old (N=21) reference
young (N=22) 1.67 (0.429 − 6.5) 0.459
Gender female (N=22) reference
male (N=21) 1.52 (0.400 − 5.8) 0.538
Stage IB−II (N=10) reference
III (N=29) 1.21 (0.234 − 6.2) 0.823
IV (N=4) 2.70 (0.184 − 39.6) 0.469
PD-L1 < 1% (N=15) reference
≥ 1% (N=28) 0.89 (0.165 − 4.8) 0.894
Adjuvant therapy no (N=20) reference
other (N=7) 0.20 (0.019 − 2.1) 0.185
radio (N=2) 3.66 (0.415 − 32.3) 0.243
targeted therapy (N=11) 0.54 (0.101 − 2.8) 0.465
Events: 13; Global p-value (Log-Rank): 0.54496
AIC: 80.68; Concordance Index: 0.68
0.02 0.05 0.1 0.2 0.5 1 2 5 10 20 50
F
Neoadjuvant Immunochemotherapy
DFS
Age old (N=21) reference
young (N=22) 1.57 (0.447 − 5.5) 0.48
Gender female (N=22) reference
male (N=21) 1.52 (0.401 − 5.7) 0.539
Stage IB−II (N=10) reference
III (N=29) 1.19 (0.232 − 6.1) 0.832
IV (N=4) 2.25 (0.122 − 41.5) 0.585
PD-L1 <50% (N=34) reference
≥ 50% (N=9) 1.17 (0.246 − 5.6) 0.84
Adjuvant therapy no (N=20) reference
other (N=7) 0.22 (0.023 − 2.2) 0.194
radio (N=2) 3.49 (0.512 − 23.8) 0.202
targeted therapy (N=11) 0.51 (0.097 − 2.7) 0.428
Events: 13; Global p-value (Log-Rank): 0.54253
AIC: 80.66; Concordance Index: 0.7
0.02 0.05 0.1 0.2 0.5 1 2 5 10 20 50
EGFR-M+ NSCLC
G
PD-L1< 50% PD-L1≥ 50%
DFS
1.00 0.75 0.50 0.25 0.00
p = 0.0015
0 12 24 36 48 60 72
Time
Number at risk
364 268 159 99 59 36 18
56 39 17 11 5 2 0
0 12 24 36 48 60 72
Time
H
PD-L1< 50% PD-L1≥ 50%
DFS
1.00 0.75 0.50 0.25 0.00
p = 0.019
0 12 24 36 48 60 72
Time
Number at risk
298 216 122 68 38 25 14
48 36 16 10 4 1 0
0 12 24 36 48 60 72
Time
