# Supplementary material for: The Role of PD-L1 in Treatment Decision-Making for Perioperative EGFR-Mutated Lung Cancer and its Genomic Analysis
Source: Ann Surg Oncol. 2026 May 27;33(10):8874–90. doi: 10.1245/s10434-026-19781-0 (PMC13586022; doi:10.1245/s10434-026-19781-0)
Supplement: Supplementary file 3 [file 10434_2026_19781_MOESM3_ESM.pdf]

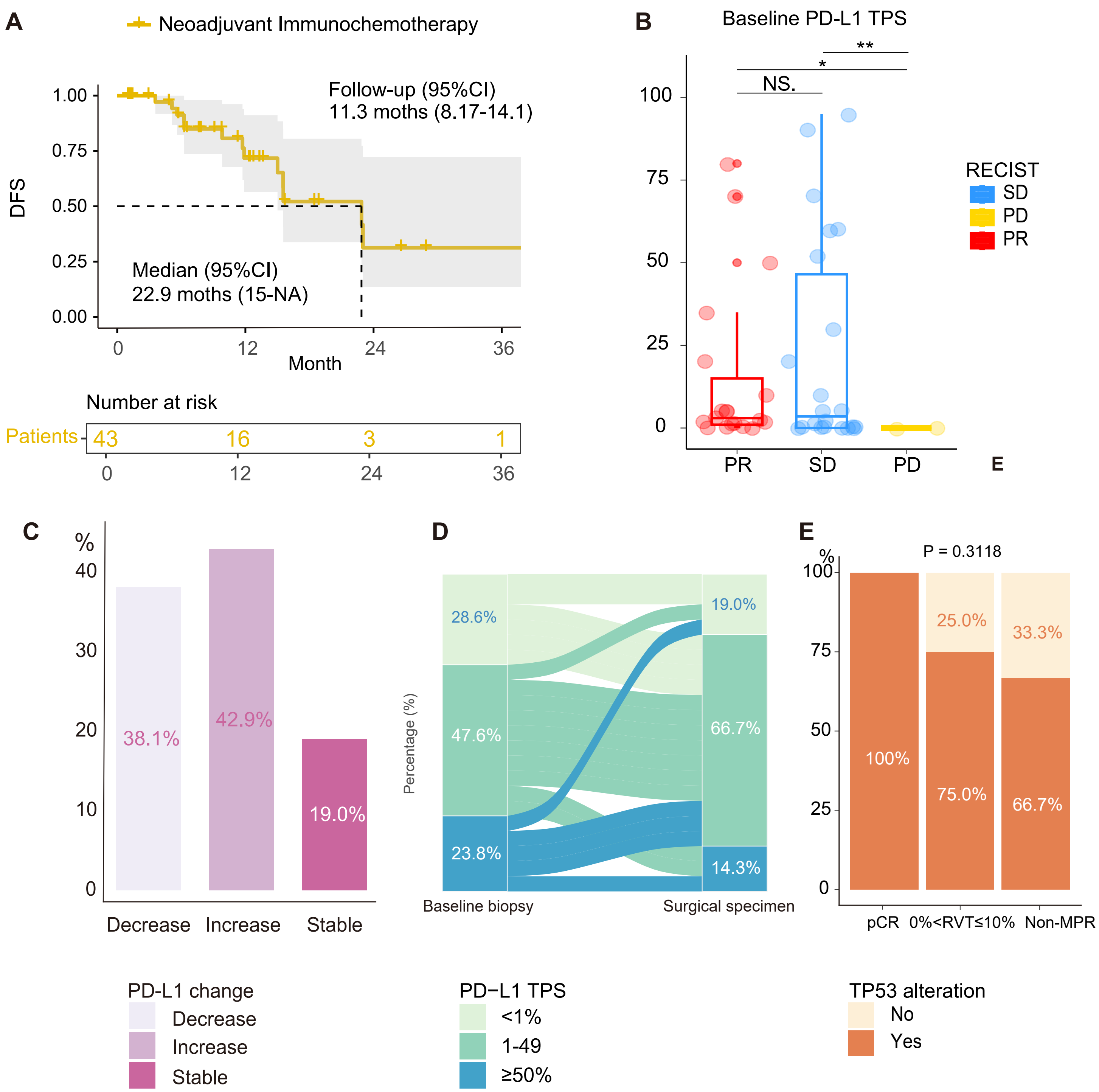
A
Neoadjuvant Immunochemotherapy
Follow-up (95%CI)
11.3 moths (8.17-14.1)
Median (95%CI)
22.9 moths (15-NA)
DFS
1.00
0.75
0.50
0.25
0.00
0
12
24
36
Month
Number at risk
Patients
43
16
3
1
B
Baseline PD-L1 TPS
NS.
*
**
100
75
50
25
0
PR
SD
PD
RECIST
SD
PD
PR
E
C
%
40
30
20
10
0
38.1%
42.9%
19.0%
Decrease
Increase
Stable
D
Percentage (%)
28.6%
47.6%
23.8%
19.0%
66.7%
14.3%
Baseline biopsy
Surgical specimen
E
%
P = 0.3118
100
75
50
25
0
100%
25.0%
75.0%
33.3%
66.7%
pCR
0%<RVT≤10%
Non-MPR
PD-L1 change
Decrease
Increase
Stable
PD−L1 TPS
<1%
1-49
≥50%
TP53 alteration
No
Yes
