# Supplementary material for: The Role of PD-L1 in Treatment Decision-Making for Perioperative EGFR-Mutated Lung Cancer and its Genomic Analysis
Source: Ann Surg Oncol. 2026 May 27;33(10):8874–90. doi: 10.1245/s10434-026-19781-0 (PMC13586022; doi:10.1245/s10434-026-19781-0)
Supplement: Supplementary file 4 [file 10434_2026_19781_MOESM4_ESM.pdf]

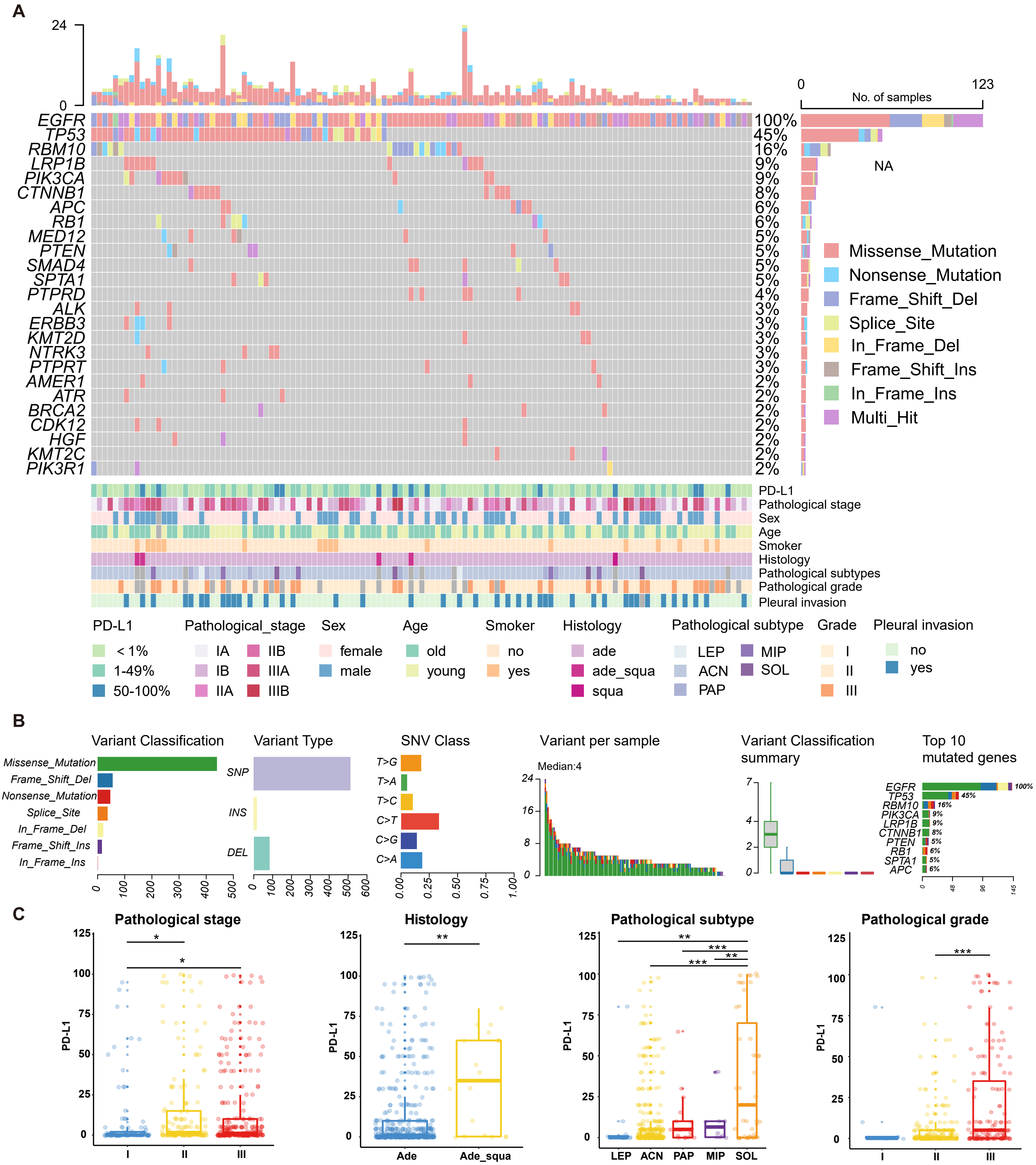
A
EGFR
TP53
RBM10
LRP1B
PIK3CA
CTNNB1
APC
RB1
MED12
PTEN
SMAD4
SPTA1
PTPRD
ALK
ERBB3
KMT2D
NTRK3
PTPRT
AMER1
ATR
BRCA2
CDK12
HGF
KMT2C
PIK3R1
100%
45%
16%
9%
9%
8%
6%
6%
5%
5%
5%
5%
4%
3%
3%
3%
3%
3%
2%
2%
2%
2%
2%
2%
2%
No. of samples
NA
Missense_Mutation
Nonsense_Mutation
Frame_Shift_Del
Splice_Site
In_Frame_Del
Frame_Shift_Ins
In_Frame_Ins
Multi_Hit
PD-L1
Pathological stage
Sex
Age
Smoker
Histology
Pathological subtypes
Pathological grade
Pleural invasion
PD-L1: < 1%, 1-49%, 50-100%
Pathological_stage: IA, IB, IIA, IIB, IIIA, IIIB
Sex: female, male
Age: old, young
Smoker: no, yes
Histology: ade, ade_squa, squa
Pathological subtype: LEP, ACN, PAP, MIP, SOL
Grade: I, II, III
Pleural invasion: no, yes
B
Variant Classification
Missense_Mutation
Frame_Shift_Del
Nonsense_Mutation
Splice_Site
In_Frame_Del
Frame_Shift_Ins
In_Frame_Ins
Variant Type
SNP
INS
DEL
SNV Class
T>G
T>A
T>C
C>T
C>G
C>A
Variant per sample
Median:4
Variant Classification summary
Top 10 mutated genes
C
Pathological stage
Histology
Pathological subtype
Pathological grade
PD-L1
Ade
Ade_squa
LEP
ACN
PAP
MIP
SOL
